# Floquet space exploration for the dual-dressing of a qubit

**DOI:** 10.1038/s41598-023-41693-2

**Published:** 2023-09-18

**Authors:** Alessandro Fregosi, Carmela Marinelli, Carlo Gabbanini, Giuseppe Bevilacqua, Valerio Biancalana, Ennio Arimondo, Andrea Fioretti

**Affiliations:** 1 Istituto Nazionale di Ottica, CNR-INO, Sede Secondaria di Pisa, Via G. Moruzzi 1, 56124 Pisa, Italy; 2 Dip. di Scienze Fisiche, della Terra e dell’Ambiente, Università degli Studi di Siena, Via Roma 56, 53100 Siena, Italy; 3 Dipartimento di Fisica, University of Pisa, Largo Bruno Pontecorvo 3, 56127 Pisa, Italy

**Keywords:** Optics and photonics, Physics

## Abstract

The application of a periodic nonresonant drive to a system allows the Floquet engineering of effective fields described by a broad class of quantum simulated Hamiltonians. The Floquet evolution is based on two different elements. The first one is a time-independent or stroboscopic evolution with an effective Hamiltonian corresponding to the quantum simulation target. The second element is the time evolution at the frequencies of the nonresonant driving and of its harmonics, denoted as micromotion. We examine experimentally and theoretically the harmonic dual-dressing Floquet engineering of a cold atomic two-level sample. Our focus is the dressing operation with small bare energies and large Rabi frequencies, where frequencies and amplitudes of the stroboscopic/micromotion time evolutions are comparable. At the kHz range of our dressed atom oscillations, we probe directly both the stroboscopic and micromotion components of the qubit global time evolution. We develop ad-hoc monitoring tools of the Floquet space evolution. The direct record of the time evolution following a pulsed excitation demonstrates the interplay between the two components of the spin precession in the Floquet space. From the resonant pumping of the dressed system at its evolution frequencies, Floquet eigenenergy spectra up to the fifth order harmonic of the dressing frequency are precisely measured as function of dressing parameters. Dirac points of the Floquet eigenenergies are identified and, correspondingly, a jump in the dynamical phase shift is measured. The stroboscopic Hamiltonian eigenfrequencies are measured also from the probe of the micromotion sidebands.These monitoring tools are appropriate for quantum simulation/computation investigations. Our results evidence that the stroboscopic phase shift of the qubit wavefunction contains an additional information that opens new simulation directions.

## Introduction

Floquet engineering has been introduced and reviewed in Refs.^1–8^. The stroboscopic component allows the realization of quantum simulations in a variety of systems, among which cold atomic samples^9–15^ and solid state ones^16–18^. In Ref.^19^ the micromotion component is considered as a limit to the measurement accuracy. On the opposite side it was investigated for an improved quantum simulation in Refs.^20, 21^, as a synthetic dimension for the characterization of topological properties in Ref.^22^, for the realization of entangled gates in Refs.^23, 24^. In the recent single dressing experiment of Ref.^25^, the resonant pumping at the micromotion evolution frequencies produced a Floquet amplification.

Bichromatic resonant driving has received a wide attention for both two- and three-level systems mainly in the resonant configurations, multiphoton and multistep, respectively. Bichromatic Fourier engineering was applied in optical lattices for the tunnelling suppression^26–28^. That driving allowed also to engineer the nearest-neighbor interactions^29^ and the dissipation processes^30^. The role of interferences in the engineering process, examined in early optical pumping experiments^31, 32^, was carefully explored in the dual modulation driving of an optical lattice clock by Ref.^33^. This reference measured also the dual dressing periodic dependence on the driving relative phase role, an issue previously theoretically investigated in Ref.^34^. Reference^35^ studied the geometric phase for the bichromatic microwave/radiofrequency dressing of colour centres. The topological features associated to an incommensurate multiple driving were theoretically explored by Refs.^34, 36^. In Ref.^37^ the dual incommensurable driving controlled evaporative cooling.

**Figure 1:**
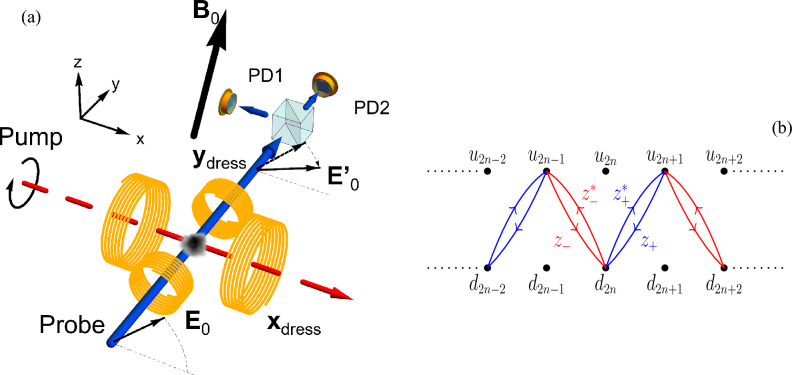
In (**a**) schematic of a qubit dressed by the $B_x$ and $B_y$ oscillating fields, generated by the X-dress and Y-dress coils, respectively, in the presence of a ${\varvec{B}}_0$ static field arbitrarily oriented in space. At $t=0$ the qubit is optically pumped into a $\sigma _x$ eigenstate by the circularly polarized pump laser propagating along the *x* axis (red dashed line). In the Resonant Pulsed Pumping mode, this pump laser stays ON, modulated at the dressed-qubit eigenfrequency (see “Methods” section). The $\langle \sigma _y(t) \rangle$ expectation value is monitored by the optical Faraday rotation of a probe beam propagating along the *y* axis (blue continuous line). Its rotation angle, from the initial $E_0$ direction to the final $E^\prime _0$ one, is analysed by a balanced polarimeter made of a polarizing beam splitter and the PD1, PD2 detectors. In (**b**) schematic representation of the Su–Schrieffer–Heeger chain of two-state sites with the complex asymmetric hopping parameters of Eq. (9). At the *n*-th horizontal site, the up/down $(u_n,d_n)$ state components are plotted with separated energies. The *n* count of the absorbed photons is equivalent to an effective force field.

The present work reports on an experimental and theoretical investigation of the global Floquet space time evolution for a cold atomic sample in a magnetometer^38^. In an external weak dc magnetic field our atomic structure is described by a collection of degenerate two-level systems. The qubit interacts with static and oscillating magnetic fields as in Fig. 1a. The qubit-field coupling is determined by the $\gamma$ constant, for a real atom being $\gamma =g\mu _B$ with *g* an effective Landé factor and $\mu _B$ the Bohr magneton, assuming $\hbar =1$. The ${\varvec{B}}_0$ static magnetic field has components $B_{0j}$ on the $j = (x, y, z)$ axes. In the dual-dressing case, the qubit is driven by two time-dependent, periodic fields oriented along the *x* and *y* axes, respectively, with $B_i$, $(i=x,y)$, maximum amplitudes. A bichromatic harmonic Hamiltonian encompassing the main features of the dual-dressing^39^ is

1 $$\begin{aligned} H =\frac{\gamma }{2} \left[ \textbf{B}_0\cdot {\varvec{\sigma }} + B_x \, \cos (\Phi _x(t)) \,\sigma _x + B_y \, \cos (\Phi _y(t)) \,\sigma _y\right] , \end{aligned}$$

with $\sigma _i$ the Pauli matrices and the $\Phi _i$, $(i=x,y)$ phases given by

2 $$\begin{aligned} \Phi _x(t)=\Phi _{0x}+\omega t, \,\quad \Phi _y(t)=\Phi _{0y}+p\omega t \end{aligned}$$

Here *p* is an integer, and $\Phi _{0i}$ with $(i=x,y)$ the initial phase of each harmonic field. The $\Delta \Phi _0=\Phi _{0y}-\Phi _{0x}$ dressing phase difference represents an additional control handle as in Ref.^33^ for the doubly-modulated optical lattice clock. The periodicity associated to the phase difference, fully equivalent to the lattice momentum periodicity in solid-state physics, represents a key element in the present investigation. In our experiment the time scale of the *x* dressing evolution corresponds to $\Phi _{0x}=0$. By taking the $\omega$ angular frequency as frequency unit in Eq. (1), we introduce dimensionless quantities as $\tau =\omega t$ time, ${\varvec{\omega }}_0 =\gamma {\varvec{B}}_0/\omega$ magnetic vector and $\Omega _i = \gamma B_i/\omega$, $(i=x,y)$ Rabi frequencies.

In the high-frequency regime the experiments of Refs.^20, 21^ investigated the micromotion at the first perturbative order in the Floquet time evolution. Here the study on strong dressing field examines experimental results associated with higher perturbation orders. While the perturbation approach of Ref.^40^ produces the physical insight into the qubit response, numerical analyses describe our experimental data. The Hamiltonian of our system is equivalent to an extended bipartite Su–Schrieffer–Heeger (SSH) model with complex tunnelling couplings depending on the dressing field phases, as pictorially represented in Fig. 1b Similar SSH models appears in one dimensional lattices with two sites per unit cell^41–45^.

In comparison with the monochromatic dressing, the dual one shows original features, as detailed in our previous publications^39, 40^. Additional original features are associated to our direct observation of the stroboscopic time evolution and of the micromotion one. In our operation this last component is not characterized by a small amplitude and a very fast timescale evolution as in previous Floquet engineering investigations. Floquet eigenenergies, eigenvalues amplitude and dynamical phase of the stroboscopic time evolution are accessed either by resonantly pumping the qubit at its eigenfrequencies, denoted as Resonant Pulsed Pumping [RPP, see “Methods” section], or by recording the time evolution after initialization, denoted as Dressed Free Evolution [DFE, see “Methods” section]. The Fast Fourier Transform [FFT, see “Methods” section] of the time evolution gives a global access to the characteristics of both stroboscopic and micromotion components. The stroboscopic spectra, i.e., the Floquet eigenenergies of the quantum simulated Hamiltonian, are measured as a function of the dressing parameters. The wide exploration of the Floquet eigenenergies complements the previous detections^17, 33, 46^. The measured Floquet quasienergy spectra vs the dressing parameters evidence the presence of Dirac points. The analysis of the stroboscopic amplitude points out the presence of parameter ranges where the detection of simulated Hamiltonian is less efficient. In the directly-monitored stroboscopic time evolution, the qubit dynamical phase shift represents an additional probe tool. The phase shift results demonstrate an interplay between the stroboscopic and micromotion components. As a surprising result we observe a discontinuity of the dynamical phase shift at the Dirac points, produced by those combined evolutions. A very good agreement between theoretical and experimental results is obtained in all examined cases.

## Results

### Dual-dressing features

#### Floquet analysis

The $U(\tau )$ time evolution operator results

3 $$\begin{aligned} i \dot{U}(\tau ) = \frac{1}{2}\left[ {\varvec{\omega }}_0\cdot {\varvec{\sigma }} + \Omega _x\, \cos (\tau +\Phi _{0x}) \,\sigma _x + \Omega _y \, \cos (p\,\tau +\Phi _{0y}) \,\sigma _y \right] U(\tau ). \end{aligned}$$

This operator is obtained numerically starting from the initial condition $U(0) = \mathbbm {1}$ and propagating until $\tau =2\pi$ using the numerical algorithm of Ref.^3^. This system is conveniently treated by the Floquet theory, a time analog of the Bloch band structure for particles in spatially periodic potentials^34^. The Floquet theorem^1–6, 8^ allows us to write

4 $$\begin{aligned} U(\tau ) = {{\,\mathrm{\displaystyle e}\,}}^{-i {\mathscr {K}}(\tau )} {{\,\mathrm{\displaystyle e}\,}}^{-i \Lambda \,\tau }. \end{aligned}$$

The qubit stroboscopic dynamics at stroboscopic times $t = n\, 2\pi /\omega$ is determined by the $\Lambda$ Floquet operator behaving as a time-independent Hamiltonian. The additional micromotion dynamics, i.e., the short time dependent evolution, is described by the ${\mathscr {K}}$ kick operator with ${\mathscr {K}}(0)=0$ and ${\mathscr {K}}(\tau +2\pi ) = {\mathscr {K}}(\tau )$. The $\Lambda$ matrix is not unique since, for a given *U* operator, one can subtract multiples of the $\omega$ frequency from its diagonal elements and compensate by adding to ${\mathscr {K}}(\tau )$ the same quantity.

We employed the numerical algorithm based on Eq. (255) of Ref.^3^ to propagate the time evolution operator from $\tau =0$ to $\tau =2\pi$. In this way we obtained $U(\tau _m)$, for $\tau _m = 2\pi \,m/N$, $m=0,1,\ldots ,N$. We checked that convergence is reached for *N* approximately few tens. The Floquet matrix is obtained as $\Lambda = (i/2\pi )\log \,U(2\pi )$ and then the kick operator ${{\,\mathrm{\displaystyle e}\,}}^{-i {\mathscr {K}}(\tau _m)}$ is obtained by inverting Eq. (4). The $\lambda _{\pm }$ Floquet eigenenergies of the $\Lambda$ single-period evolution operator are restricted to the $(- 0.5,0.5)$ first Brillouin zone. The $\Lambda$ matrix may be written as

5 $$\begin{aligned} \Lambda = \frac{1}{2} \, {\varvec{h}} \cdot {\varvec{\sigma }}, \end{aligned}$$

where the ${\varvec{h}}$ vector, measured in energy units, represents an effective magnetic field. From a quantum simulation point of view, the stroboscopic response of a dual-dressing qubit is described by the ${\varvec{h}}$ magnetic field arbitrarily oriented in space with a maximum absolute value determined by the dressing frequency^40^. In the experimental investigation the $\Lambda$ Floquet eigenenergies associated to the $(|\lambda _+\rangle ,|\lambda _-\rangle )$ eigenvectors are characterized by the following $\Omega _L$ dressed Larmor frequency:

6 $$\begin{aligned} \Omega _L =|\lambda _+ - \lambda _-| = |{\varvec{h}}|. \end{aligned}$$

**Figure 2:**
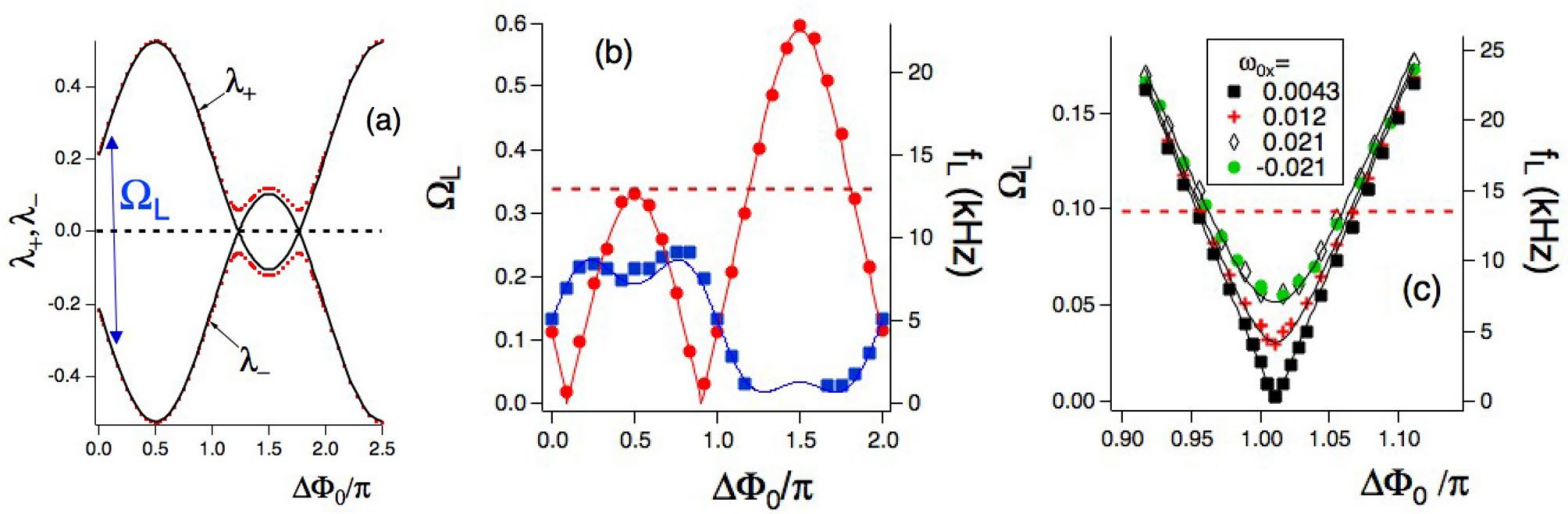
$\Delta \Phi _0$ dependence of theoretical $(\lambda _+,\lambda _-)$ Floquet eigenenergies and $\Omega _L$ dressed frequency, and of measured $f_L$ frequencies. In (**a**) $(\lambda _+,\lambda _-)$ with the *x* axis larger than the first Floquet zone to evidence the eigenenergy periodicity. $\Omega _L$ is derived from the $|\lambda _+-\lambda _-|$ difference. Parameters: $p=1$, $\Omega _x=1.3, \Omega _y=0.64$, $\omega _{0z}=0.3375$ and $\omega _{0y}=0$. Black line for $\omega _{0x}=0$, and red dots for $\omega _{0x}=0.03$. In (**b**) $\Omega _L$ theory (continuous lines) and $f_L$ experiment (markers) dressed Larmor frequencies within the Floquet zone. Blue line and squares for $\Omega _{x}=2.60(1)$ and $\Omega _{y}=3.15(2)$, red line and circles for $\Omega _{x}=2.60(1)$ and $\Omega _{y}=1.90(1)$. Other parameters: $p=1$, $\omega _{0x}=\omega _{0y}=0$, $f=40.00\,$ kHz, $\omega _{0z}=0.3375(3)$ corresponding to $f_0=13.50(5)\,$ kHz. In (**c**) $\Omega _L$ and $f_L$ within a limited Floquet zone. The transition from a Dirac-like crossing into an anti-crossing is explored by tuning the $\omega _{0x}$ field. Parameters: $p=1$, $\Omega _{x}=1.670(5)$, $\Omega _{y}=1.20(1)$, $f=135.00\,$ kHz $\omega _{0y}=0$, $\omega _{0z}=0.100(3)$, corresponding to $f_0=13.50(5)\,$ kHz. In both experimental plots the error bar is smaller than the dot size, and the red dashed horizontal lines denote the $f_0=13.50(5)\,$ kHz undressed Larmor frequency value. All experimental data are acquired in the RPP mode. The number in brackets represents, according to the standard notation, the error in the last digit.

Figure 2a reports theoretical $\Lambda$ eigenvalues vs the $\Delta \Phi _0$ dressing phase for fixed $\Omega _x,\Omega _y$ amplitudes. Zero-crossing points, i.e., Dirac-like points, appear for specific dressing parameter values. The eigenvalues may reach the Brillouin zone with crossings modified into anti-crossings. The $(\lambda _+,\lambda _-)$ symmetry shown in Fig. 2a leads to $|\lambda _+|= |\lambda _-|=\Omega _L/2$. The $\Omega _L$ maximum value is 1, i.e., the dressing frequency.

#### Quantum simulation and SSH analogy

References^33, 34, 47^ pointed out the strong analogy between time-periodic Hamiltonians, as the Eq. (1) one, and tight-binding models in presence of a static electric field and with each lattice site coupled to its neighbors. For such a comparison the action of the $U(\tau )$ operator on a $|\lambda \rangle$ eigenstate is written as

7 $$\begin{aligned} U(\tau ) | \lambda \rangle = {{\,\mathrm{\displaystyle e}\,}}^{-i {\mathscr {K}}(\tau )} | \lambda \rangle {{\,\mathrm{\displaystyle e}\,}}^{-i \lambda \tau } \equiv | \lambda (\tau ) \rangle {{\,\mathrm{\displaystyle e}\,}}^{-i \lambda \tau }. \end{aligned}$$

The $|\lambda (\tau )\rangle$ time periodic structure is given by

8 $$\begin{aligned} | \lambda (\tau ) \rangle = \sum _n P_n | \lambda \rangle {{\,\mathrm{\displaystyle e}\,}}^{i n\tau } \equiv \sum _n \begin{pmatrix} u_n\\ d_n \end{pmatrix} {{\,\mathrm{\displaystyle e}\,}}^{i n\tau }, \end{aligned}$$

with $(u_n,d_n)$ the components of the $P_n|\lambda \rangle$ state vector associated to *n* dressing photons [for the $P_n$ operators see Eq. (3) of the Supplemental Information^48^]. Substituting Eqs. (8) and (7) in Eq. (3), after some algebra we obtain the following coupled recurrences (valid for the $p=1$ case only):

9a $$\begin{aligned} \lambda u_n&= (n + \frac{\omega _0}{2}) u_n + \frac{1}{4}( \Omega _x - i\Omega _y{{\,\mathrm{\displaystyle e}\,}}^{-i \Delta \Phi _0} )d_{n+1} + \frac{1}{4}( \Omega _x - i\Omega _y{{\,\mathrm{\displaystyle e}\,}}^{i \Delta \Phi _0} )d_{n-1}, \end{aligned}$$

9b $$\begin{aligned} \lambda d_n&= (n - \frac{\omega _0}{2}) d_n + \frac{1}{4}( \Omega _x + i\Omega _y {{\,\mathrm{\displaystyle e}\,}}^{-i \Delta \Phi _0} )u_{n+1} + \frac{1}{4}( \Omega _x + i\Omega _y {{\,\mathrm{\displaystyle e}\,}}^{i \Delta \Phi _0} )u_{n-1}, \end{aligned}$$

where, for simplicity, we used ${\varvec{\omega }}_0= (0,0,\omega _{0z})$. These equations describe a tight-binding chain with two states per site, i.e., a dimerized chain, (see Fig. 1b for a representation) with the following complex asymmetric nearest-neighbour hopping strengths:

10 $$\begin{aligned} z_{\pm } =\frac{1}{4}( \Omega _x - i\Omega _y{{\,\mathrm{\displaystyle e}\,}}^{\pm i \Delta \Phi _0} ). \end{aligned}$$

For $\Delta \Phi _0 = \pm \pi /2$ the $z_{\pm }$ hopping parameters reduce to $(\Omega _x \pm \Omega _y)/4$, with the above periodic structure derived in Ref.^1^. For $\Omega _y=0$ the above equations reduce to the Wannier-Stark model as in Refs.^34, 49^. Within Eqs. (9a) and (9b) the top(bottom) $u_n(d_n)$ state has an intrinsic potential energy $\omega _0/2(-\omega _0/2)$. The $n u_n$ and $n d_n$ terms represent the interaction with an effective electric field force associated to the photon number as in Ref.^34^ for an incommensurate dual-dressing. The recurrence structure of Eq. (9a) and (9b) reveals a chain internal decoupling. The even-position bottom states are coupled only to the odd-position top states, leading to a chain with only one state per site. Similarly the odd-position bottom states couple only to the even-position top states, producing a separate chain of one state per site. The coupling between these two chains is produced by the qubit preparation in any superposition of top and bottom states. For $p \ne 1$ one obtains a similar chain structure based on two coupled recurrences with $\Omega _x$ nearest-neighbour hooping and a complex hopping between the sites *n* and $n \pm p$ due to $\Omega _y$. The structure of all these chains recall the SSH model, with the additional presence of the photon number force. Except for this parameter, direct analogies exist between Eq. (9a) and (9b) and similar ones for the complex amplitudes of a bipartite lattice based on chain of optical resonators^42^. A similar analogy exists also for the extended SSH models based on two sublattices of Refs.^41, 43–45^, with the photon force replaced by hopping strengths. For the Floquet engineering of a driven two-band system Ref.^50^ pointed out the analogy with an SSH model including additionally nearest-neighbor interactions.

### Probing the stroboscopic evolution

The stroboscopic time periodic evolution is determined by the $\Lambda$ eigenvectors and the associated $\Omega _L$ dressed Larmor frequencies. In the experiment, the $\langle \sigma _y(\tau ) \rangle$ mean value of the qubit spin is monitored as in Fig. 1a, following its initial preparation in the $\sigma _x(0)=1$ state [see Setup in “Methods” section]. Using the RPP probe, the $f_L$ experimental dressed Larmor frequency is measured, and compared to the theoretical $\Omega _L$ one. Data for the Floquet eigenenergy dependence on the $\Delta \Phi _0$ relative dressing phase and on the dressing Rabi frequencies are collected. An additional stroboscopic information is given by the amplitude of the $\langle \sigma _y \rangle _{\Omega _L}$ qubit oscillation at the dressed $(\Omega _L,f_L)$ frequency.

#### Phase periodicity of Floquet eigenvalues

For a time-periodic Hamiltonian the quasi momentum periodicity in solid state is replaced by a periodicity in the dressing field phase. For a bichromatic time-periodic Hamiltonian, the periodicity is associated with the ${\varvec{\Phi }}=(\Phi _x,\Phi _y)$ phase vector of Eq. (1), as pointed out in Ref.^34^ treating the periodic energy exchange between the two driving fields. The periodic time evolution is described by a closed orbit in the 2D $(\Phi _x,\Phi _y)$ space. In analogy to the solid state case, we refer to the regions $0 \le \Phi _{x0},\Phi _{y0} \le 2\pi$ of initial phases as the Floquet zones^34^. The theoretical data of the Floquet eigenenergies of Fig. 2a for fixed $\Omega _x,\Omega _y$ amplitudes evidence the periodic dependence as a function of the $\Delta \Phi _0$ relative dressing phase. The strong dependence on the $(\Phi _{x0},\Phi _{y0})$ initial values derived in Ref.^34^ applies also to our $\Delta \Phi _0$ dependence.

For given $\Omega _x,\Omega _y$ dressing fields, we verify experimentally the phase periodicity of the Larmor frequency and its fine tuning through the $\Delta \Phi _0$ control parameter. Figure 2b reports the measured $f_L$ frequency vs $\Delta \Phi _0$ in the Floquet zone for two sets of dressing strengths, at given $f=\omega /2\pi$ experimental dressing frequency and $f_0=|{\varvec{\omega }}_0|/2\pi$ undressed frequency. $\Omega _L$ maxima and minima appear by tuning the dressing phase difference, as derived from the theory eigenvector lines of Fig. 2a. They appear also in theory/experiment data of Fig. 2b.

**Figure 3:**
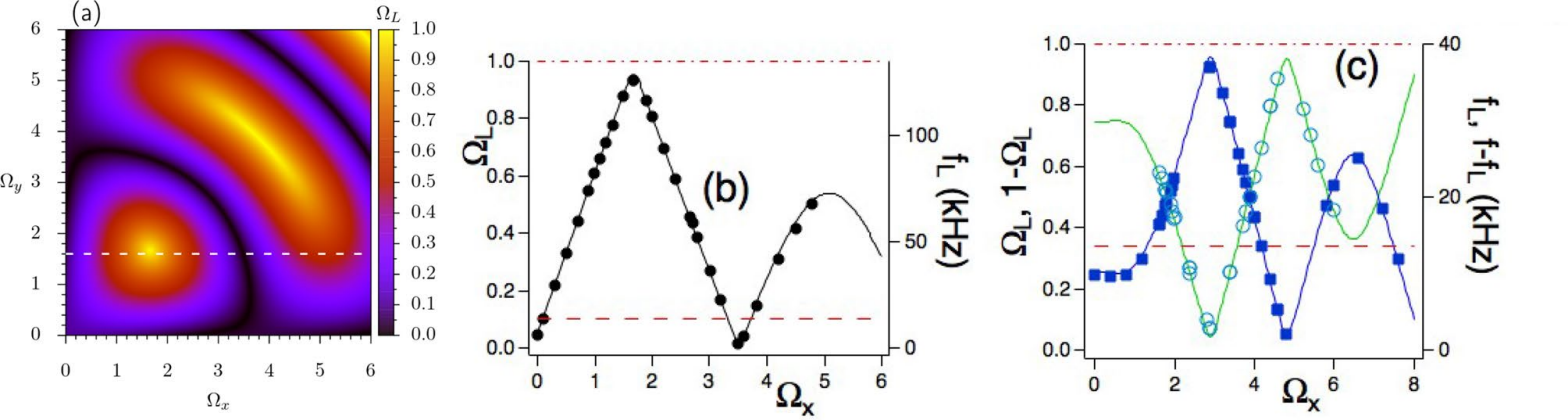
Theoretical $\Omega _L$ and experimental $f_L$ Larmor frequencies vs Rabi dressing frequencies. In (**a**) 2D $(\Omega _x,\Omega _y)$ map of $\Omega _L$ for $p=1$, $\Delta \Phi _0/\pi =0.5$, and $\omega _{0z}=0.1$. In (**b**) theoretical prediction (line) and experimental data (markers) for dressed Larmor frequencies vs $\Omega _x$. The theoretical prediction corresponds to the horizontal white line of the (**a**) plot. Experimental parameters: $\Omega _y=1.60(1)$, $\omega _{0z}=0.100(3)$, $\Delta \Phi _0/\pi =0.500(5)$, and $f=135.00\,$ kHz. In (**c**) $\Omega _L$ theory (blue line) and $f_L$ experiment (markers) for the $p=2$ case vs $\Omega _x$. Green line and open dots for $1-\Omega _L$ and $f-f_L$ micromotion sideband, see text. Parameters: $\Omega _y=2.00(1)$, $\Delta \Phi _0/\pi =0.00(1)$ and $f=40.00\,$ kHz. In both experimental plots the error bar is smaller than the dot size. In (**b,c**) the horizontal red dot-dashed lines indicate *f* values while dashed lines $f_0$ ones. In all plots $\omega _{0x}=\omega _{0y}=0$, and undressed Larmor frequency $f_0=13.50(5)\,$ kHz. All data in (**b**) and the blue ones in (**c**) are collected in the RPP mode.

#### Floquet eigenenergies vs Rabi frequencies

For the Floquet eigenenergies vs dressing strengths, Fig. 3a shows the theoretical 2D $(\Omega _x,\Omega _y)$ map of $\Omega _L$, at given $\Delta \Phi _0$ value and $p=1$, with maxima and minima. Figure 3b reports $\Omega _L$ theoretical and $f_L$ experimental data vs $\Omega _x$ at $\Omega _y=1.45$ and $p=1$, corresponding to the horizontal dashed white line of Fig. 3a. As from the theory map, at $\Omega _x=1.685$ and $\omega _{0z}=0.1$ the $\Omega _L\approx 0.965$ dressed Larmor frequency approaches the upper boundary zone. This response corresponds to a substantial increase of almost one order of magnitude for the qubit Larmor frequency, for both $(\Omega _L,f_L)$ data. Similar results are obtained for the $p=2$ case, as in Fig. 3c reporting $f_L$ experimental results, filled squares, and $\Omega _L$ theoretical ones, blue continuous line, vs $\Omega _x$ at fixed $\Omega _y$ dressing. Such similarity applies to all *p* values of the double-dressed Hamiltonian. The green open circles and continuous line of that figure are discussed in the following global probe section.

#### Crossings, anticrossings, and Dirac points

The 2D map of Fig. 3a reports $(\Omega _x,\Omega _y)$ values where $\Omega _L=1$, i.e., the Floquet eigenenergies reach the Brillouin zone boundary leading to crossing points at its bottom and top. These crossings are transformed into anti-crossings for different values of the dressing strengths. The anticrossing maxima are present in the $\Omega _L$ plots vs the dressing strengths of Fig. 3b. In the $p=1$ plot of Fig. 2b, the $(\Omega _L,f_L)$ maxima appear at the $\Delta \Phi _0/\pi =0.5$ and 1.5 values where the dressing field is composed by rotating and counter-rotating components, respectively, strongly coupled to the qubit at our very low static magnetic fields. The $\omega _{0z}$ amplitude modifies the coupling strength, and in consequence the $\Omega _L$ maxima values.

Dirac-like points, i.e., zero values of the $\Lambda$ eigenenergies and the $\Omega _L$ frequency, appear by tuning the dressing field phases, as in plots of Fig. 2, or by tuning the dressing strengths, as in Fig. 3b,c. The two Dirac-like points appearing in $(\Omega _L,f_L)$ vs $\Delta \Phi _0$ (theory black line of Fig. 2a and red line one of Fig. 2b, with experimental data red dots) have positions that depend on the dressing parameters and are destroyed, i.e., transformed into anticrossings, by increasing the dressing amplitude. The blue line and squares of Fig. 2b evidence such destruction.

We have an additional handle for such crossing-anticrossing transformation. This handle is a weak transverse magnetic field, either $\omega _{0x}$ or $\omega _{0y}$, as shown in the red dotted theoretical line for $(\lambda _+,\lambda _-)$ vs of Fig. 2a. This transformation is examined experimentally, for the zero crossing, in the $(\Omega _L,f_L)$ vs $\Delta \Phi _0$ plot of Fig. 2c, which reports Larmor frequency values in a limited Floquet zone range around one Dirac point. With an applied $\omega _{0x}$ static magnetic field scanned around the $\omega _{0x}=0$ compensation value, the crossing-anticrossing transformation is carefully investigated. The dashed lines report the theoretical predictions.

**Figure 4:**
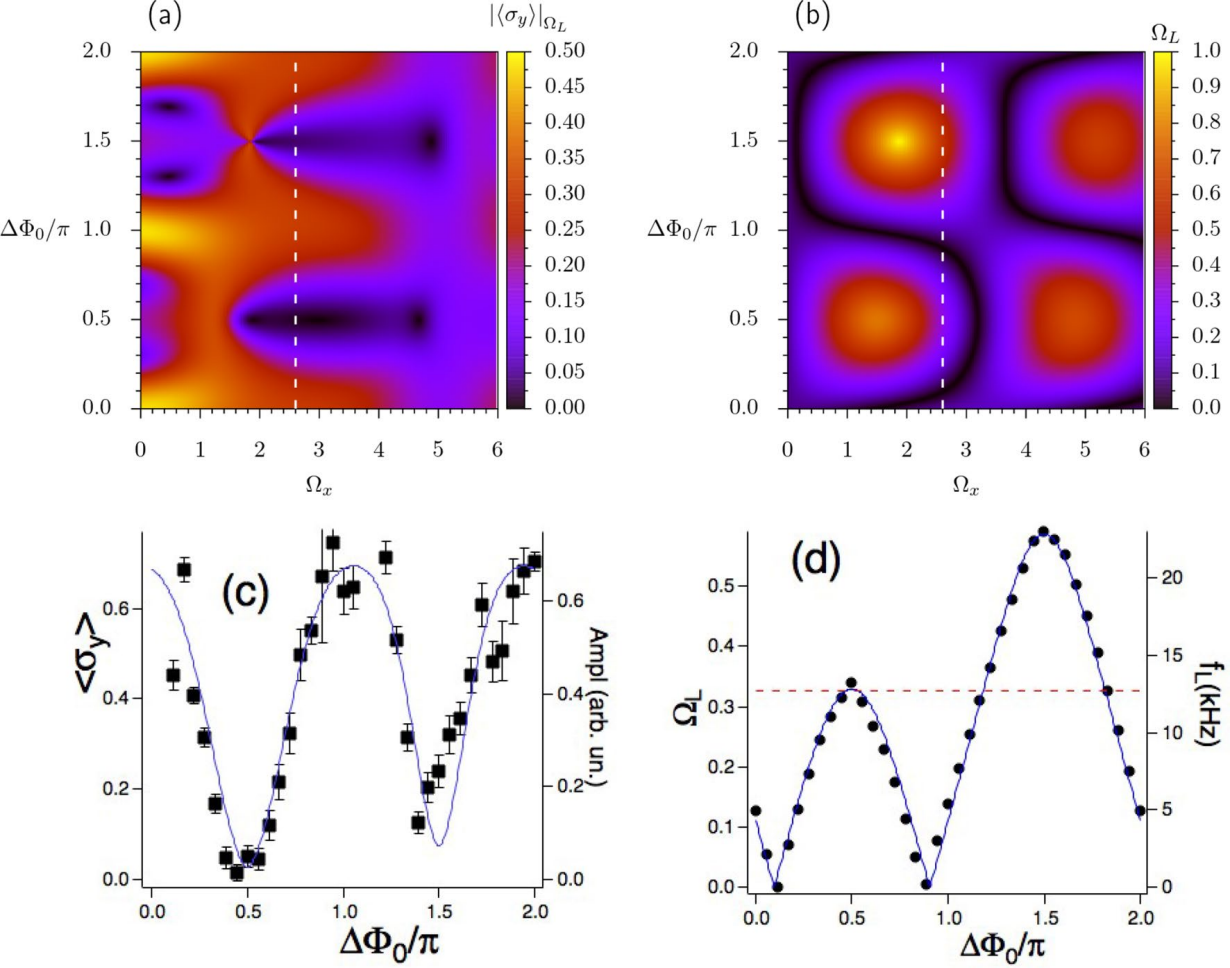
The $|\langle \sigma _y\rangle |_{\Omega _L}$ stroboscopic component amplitude at the $\Omega _L$ frequency, and analysis of the corresponding Larmor frequency . In (**a,b**) 2D theoretical maps in the $(\Omega _y,\Delta \Phi _0)$ plane; in (**a**) for the $|\langle \sigma _y\rangle |_{\Omega _L}$ stroboscopic component amplitude at the $\Omega _L$ frequency; in (**b**) for the $\Omega _L$ frequency. Colour scales on the right. In (**c,d**), measured $|\langle \sigma _y\rangle |_{\Omega _L}$ amplitude and $f_L$ Larmor frequency vs $\Delta \Phi _0$, respectively. $f_L$ error bars are smaller than the dot size. Theoretical predictions correspond to the white vertical cuts in the (**a,b**) 2D maps. Parameters in bottom plots: $p=1$, $\Omega _x = 2.60(1), \Omega _y = 1.90(1)$, $\omega _{0x}=\omega _{0y}=0$, $\omega _{0z}=0.3375(3)$. In (**c,d**) $f=37.90\,$kHz and $f_0=12.70(5)\,$kHz, denoted in (**d**) by the red dashed line.

**Figure 5:**
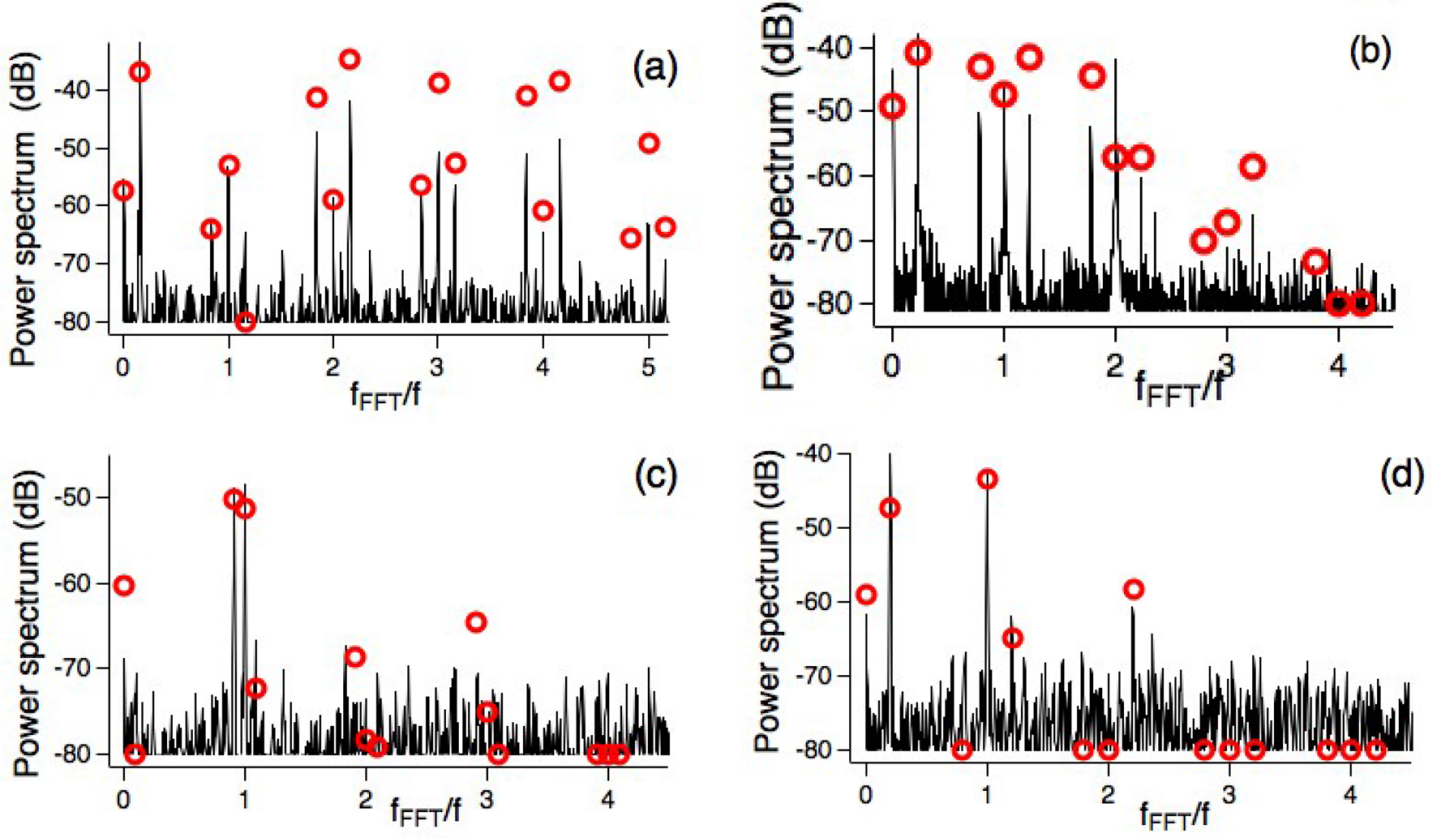
FFT power spectrum (continuous black line, arbitrary units) of $\langle \sigma _y(\tau ) \rangle$ vs $f_{\textrm{FFT}}$ Fourier frequency, measured in *f* units. All spectra obtained from FFT applied to the RPP signals. The *s*-th micromotion component appears at $f_{\textrm{FFT}}=s$ with its sidebands at $s\pm \Omega _L$ positions. When the sidebands are stronger than the main components, the regular spacing of the micromotion components at first glance does not appear satisfied. Red open circles represent theoretical predictions scaled to the experimental ones. Power spectrum measured in arbitrary units. On the low power values the experimental and theoretical spectra are limited to the − 80 dB value.The $0, \Omega _L,1, 2,3,...$ peak sequence, with their ordered micromotion sidebands, appears in the (**a,b,d**) plots. Instead in (**c**) the $\Omega _L$ large value leads to the $0,1- \Omega _L, \Omega _L, 1, 2-\Omega _L,1+\Omega _L, 2, 3- \Omega _L,..$ sequence. Parameters $[p,\Omega _x,\Omega _y,\Delta \Phi _0/\pi ,f_L]$ the last one in kHz: in (**a**) [1, 4.00(1), 0.850(4), 0.500(1), 6.4(1)]; in (**b**) [2, 1.400(4), 0.550(3), 0.000(1), 8.8(1)]; in (**c**) [1, 1.340(4), 1.380(7), 0.500(1), 36.5(1)]; in (**d**) [1, 1.000(3), 1.000(5), 1.500(1), 8.0(1)]. In all plots $\omega _{0x}=\omega _{0y}=0$, $\omega _{0z}=0.3375(3)$, and $f=40.00$ kHz.

#### Amplitude of the stroboscopic oscillation

The $\langle \sigma _y(t)\rangle _{\Omega _L}$ amplitude oscillation at the $\Omega _L$ frequency is examined under different operating conditions. It has a complex dependence on the dressing parameters as in theoretical 2D $(\Omega _x,\Omega _y)$ map of Fig. 4a. The 2D plot of Fig. 4b reports the corresponding $\Omega _L$ values. Experimental results for the white lines vertical cuts of the 2D maps of Fig. 4a,b are presented in (c,d). In contrast to the excellent experiment-theory matching for the dressing frequencies, for the amplitude only a good agreement is reached. Note that in this case the precise alignment of the probe with the *y* axis is a critical issue. A comparison of the theory/experiment plots evidences that the amplitude of the stroboscopic component is not constant, and is greatly depressed for dressing parameters close to the $(\Omega _L,f_L)$ maxima. Such different response becomes important when the stroboscopic simulated Hamiltonian is explored experimentally.

### Probing the global Floquet space

The exploration of the Floquet space is completed by measuring the time dependence of the global qubit evolution. For the measured $\langle \sigma _{y}(\tau ) \rangle$ qubit component, such global evolution is described by Eq. (12) [see Qubit evolution in “Methods” section]. The key feature is the presence of different time scales: a time periodic evolution at the $\Omega _L$ dressed Larmor frequency, superimposed on the micromotion evolution at the *s*-th harmonic of the dressing frequency, with *s* an integer, and, finally, the $s \pm \Omega _L$ dressed-frequency micromotion-sidebands. They are experimentally monitored by all probes.

#### Micromotion sideband frequencies

As in the previous Larmor frequency subsection, we measure the frequencies of the micromotion sidebands applying the RPP probe to the qubit $$\langle \sigma _y(\tau ) \rangle$$ at the *s*-th sideband frequency. For the low frequency sideband of the $$s=1$$ micromotion component, the $$1-\Omega _L$$ theoretical predictions, and the $$f-f_L$$ experimental results, are plotted in Fig. 3c vs $$\Omega _x$$ at fixed $$\Delta \Phi _0$$ and $$\Omega _y$$ values. The comparison of those data with the $$(\Omega _L,f_L)$$ ones within the same figure confirms that the frequency of the micromotion sideband at $$(1-\Omega _L$$, $$f-f_L)$$, represents a mirror image of the $$\Omega _L$$ theory frequency, $$f_L$$ in the experiment. The information on the quantum simulated Hamiltonian is stored also in the micromotion time evolution.

#### FFT exploration

Figure 5 reports FFT spectra recorded for several dressing conditions, with micromotion frequency components at the multiples of the *f* dressing frequency up to the fourth harmonic, and their $f\pm \Omega _L$ sidebands. Note the presence of the zero frequency component in all spectra, described theoretically by the first line of Eq. (12). In Fig. 5c the large value of the $\Omega _L$ frequency, close to the 1 maximum value, leads to sidebands largely shifted from each micromotion harmonic component. The theoretical predictions for the FFT spectrum peaks based on numerical analyses provide a good match of the experimental observations, the higher order micromotion components being depressed in amplitude by the detection bandwidth. Owing to the interferences in the qubit response, the relative amplitude of the spectrum components has a strong dependence on the dressing parameters. For instance, in the Fig. 5c,d plots one sideband is significantly weaker than the other. The theoretical simulations pointed out an interesting qubit response while monitoring the components of the qubit spin along the *x*, *z* directions. For instance, on the $\langle \sigma _z(t)\rangle$ time evolution, the odd micromotion components do not appear in the spectra owing to their reduced amplitude. Such simplified dressed qubit response represents a configuration useful for the probe issues in quantum simulation.

#### Time exploration

**Figure 6:**
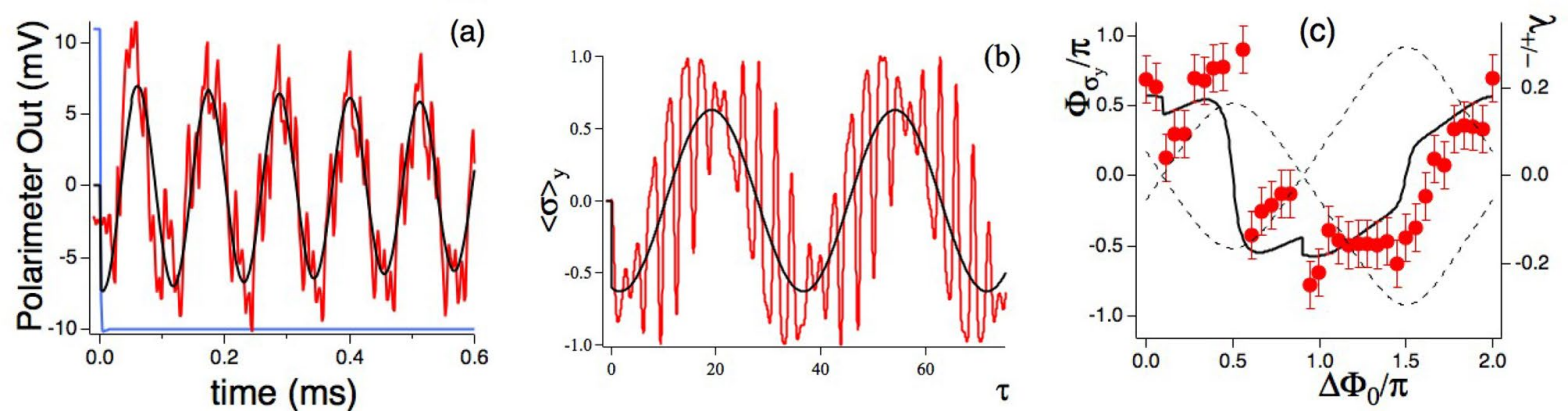
Experiment and theory $\langle \sigma _y(\tau ) \rangle$ following the $t=0$ switching of the dressing fields, and the qubit $\Phi _{\sigma _y}$ measured/theory phase. In (**a**) experimental $\langle \sigma _y(\tau ) \rangle$ (red line) for $\Delta \Phi _0/\pi =1.220(2)$, $\Omega _x=2.600(8),\Omega _y=1.90(1)$. The blue line denotes the trigger for the dressing switch. In (**b**) corresponding theoretical prediction. Black lines on both plots represent sinusoidal fits based on Eq. (11). The $f_L,\Omega _L$ value is determined independently, see text. The derived phase shifts are $\Phi _{\sigma _y}/\pi = - 0.58(11)$ measured and $\Phi _{\sigma _y}/\pi =-0.5197$ theory. In (**c**) $\Phi _{\sigma _y}$ measured (red dots with error bar) and theoretical (black line) phases of $\Omega _L$ oscillations vs the $\Delta \Phi _0$ dressing phase. Experimental data derived from the DFE exploration. On the right axis the theoretical $\lambda _{\pm }$ Floquet eigenenergies vs $\Delta \Phi _0$. The sharp variations of $\Phi _{\sigma _y}$ occur at the Dirac points. A $\approx \pi$ phase change occurs around the $|\lambda _{\pm }|$ maxima.

The observation of the $\langle \sigma _y(\tau ) \rangle$ time evolution provides a direct access to the combined stroboscopic and micromotion components. It represents a precise probe of their relative contribution to the total qubit response. The $\langle \sigma _y(\tau ) \rangle$ time evolution is monitored following the switch-on of the dressing fields at the initial $t=\tau =0$ time [see Dressed free evolution (DFE) in “Methods” section]. Experimental data and theoretical simulations are presented in Fig. 6a,b, respectively. Those time evolutions clearly evidence the $\Omega _L$ precession and the micromotion oscillations owing to their different frequencies for the chosen dressing parameters. The Larmor amplitude, greater than the micromotion one, is described by $\Omega _L$ sinusoidal fits, black lines in the plots. The micromotion components at the first and second harmonic frequencies are directly identified on both plots. Their relative amplitude depends greatly on the dressing parameters, as presented in the previous FFT exploration Subsection.

#### Qubit phase shift at $t=0$

The qubit evolutions of Fig. 6a,b present a very interesting feature at the short $(t,\tau \approx 0)$ times. On the basis of the second line of Eq. (12) within the Qubit Evolution [see “Methods” section], the $\Omega _L$ stroboscopic component of the qubit $\sigma _y$ is written as

11 $$\begin{aligned} \langle \sigma _y(t) \rangle _{\Omega _L}\propto \sin (\Omega _L t +\Phi _{\sigma _y}), \end{aligned}$$

where we introduce a $\Phi _{\sigma _y}$ dynamical phase shift produced by the micromotion evolution. Note that in magnetic resonance experiments with a single rotating dressing field and starting from $\langle \sigma _x(0)\rangle =1$, the $\langle \sigma _y(0)\rangle =0$ initial condition leads to $\Phi _{\sigma _y}=0$. This applies also to the single dressing time evolution as derived in Ref.^39^. For an incommensurate dual driving Ref.^35^ linked the $\Phi _{\sigma _y}$ phase shift to a high order geometric phase.

The black lines of Fig. 6a,b report sinusoidal fits based on Eq. (11) with $f_L$ values derived from the Larmor frequency measurements as in Figs. 2 and 3 for the experimental data, and from the numerical eigenvalue determinations for the $\Omega _L$ theoretical ones. From those fits we derive that the $\Phi _{\sigma _y}(0)=0$ condition valid for magnetic resonance and single dressing does not apply. The theoretical simulations evidence that the $\langle \sigma _y(t \approx 0) \rangle \approx 0$ continuity is satisfied by the micromotion evolution, as from a close exam of the $\tau =(0,2)$ data in Fig. 6b. The $\Omega _L$ oscillations begin with a phase shift different from zero in both Fig. 6a,b.

Fits of the experimental time evolutions on the basis of Eq. (11) at given dressing field amplitudes produce the $\Phi _{\sigma _y}$ vs $\Delta \Phi _0$ plot of Fig. 6c. The theoretical counterpart (black continuous line of Fig. 6c) is obtained by computing the phase from the second line of Eq. (12). The dashed line there reports the associated $\lambda _{\pm }$ dependence on $\Delta \Phi _0$, similar to the theoretical result of Fig. 2a. A smooth and large $\pi$ phase shift takes place at the anticrossings points corresponding to the $|\lambda _{\pm }|$ maxima. At the $\lambda _{\pm }=0$ Dirac-like points a sharp and small, approximately $\pi /10$, discontinuity of the $\Phi _{\sigma _y}$ phase shift takes place. The amplitude of the $\Phi _{\sigma _y}$ sharp jump at the Dirac-like points is modified by the dressing parameters. Instead the amplitude of the smooth and large $\pi$ phase change around the $|\lambda _{\pm }|$ maxima depends weakly on those parameters. For a theoretical connection between the $|\lambda _{\pm }|$ linear variation at the Dirac points and the phase shift jump see Phase shift discontinuity in Supplemental Information^48^. The comparison of the measured/predicted $\Omega _L$ and $\Phi _{\sigma _y}$ values at the $\Delta \Phi _0=0$ and $\Delta \Phi _0/\pi =1$ dressing phases for fixed dressing amplitudes represents an interesting issue. At those phases the dressing fields have the same geometry, except for a modified spatial orientation. This symmetry leads to an equal $\Omega _L$ Larmor frequency for those phases as shown by the data in Figs. 2b and 4d. Instead the results of Fig. 6c evidence a $\pi$ change of the $\Phi _{\sigma _y}$ value at those dressing phases.

## Discussion

The present work explores the stroboscopic and micromotion components of the qubit dynamics in the Floquet engineering of two-level cold atoms released from a magneto-optical trap. The qubits are based on the ground-state low-field magnetic field splitting. Our Hamiltonian includes two harmonic radiofrequency magnetic interactions. We operate in a regime of small undressed energy splitting and large dressing Rabi frequencies, larger than the Floquet dressing frequency. The low frequency operation represents a key element for our diagnostic tools. The diagnostics is based on ad-hoc probes. The micromotion components and their sidebands appearing in the FFT and in the time-evolution signals provide a clear insight into the global qubit dynamics. By tuning the RPP frequency we examine separately the stroboscopic and micromotion components of the Floquet space evolution.

Dirac-like points are present in the stroboscopic spectra of the dual-dressed system vs the dressing phase difference. At the Dirac points we observe a phase-discontinuity for the stroboscopic qubit evolution. Its dependence on the dressing parameters represents a peculiar signature. It will be important to explore if such signature applies also to Dirac points in other quantum systems, for instance, in the SSH models with a structure similar to Fig. 1b. The connection with the shift in the Berry’s phase associated to the Dirac points examined, for instance, in Ref.^51^ should be also explored. In Ref.^52^ for Dirac points not isolated in space a nodal structure was introduced as a topological invariant, whose form depends on their symmetry group. This approach should be applied also to the Dirac lines appearing in the $(\Omega _x,\Omega _y)$ space of our dual-dressing.

The present experiment is limited by the interaction time of the cold atoms. Longer interaction times are obtained by confining the atoms in an optical trap. Those times are required for a test of the Berry phase or the Chern number in the double-dressing with incommensurable frequencies.

From the points of view of quantum control and quantum simulation, our study of the stroboscopic and micromotion components produces several results to be exploited in future Floquet engineering investigations. The produced effective magnetic field arbitrarly controlled in orientation and amplitude, should be applied to qubit experiments requiring an easy and adiabatic control of the spin orientation. The qubit pulsed pumping in a strong regime is equivalent to a parametric excitation, and this connection may represent an additional handle in the Floquet engineering. The observations of the phase shift and its discontinuity evidence that in quantum simulations the phase shift of qubit wavefunction contains an additional information opening new simulation directions. The role of the driving dressing phases in the multifrequency Floquet engineering should be also examined within the same context. We apply the RPP to the exploration of qubit evolution for both the stroboscopic component and the micromotion ones, at the dressing harmonics and their sidebands. Such direct and precise determination of the micromotion time evolution opens the road to a quantum control application. A parametric driving of the micromotion components is equivalent to the storing of the additional information in our qubit. Within this approach the micromotion components play the role of synthetic dimensions and become an additional quantum control handle for the Floquet engineering.

While our attention is focused on single qubit system, it will be interesting to investigate the dual-dressing features also in presence of interaction and relaxation precesses. For this last topic the existence of periodic steady-state independent of the initial conditions was already proven in Ref.^53^.

## Methods

### Qubit evolution

The dressing operation modifies mean value and time evolution of the spin components. These quantities are derived from the $U(\tau )$ operator time evolution of Eq. (4) using the ${\mathscr {K}}$ kick operator and the $\Lambda$ stroboscopic one. From Eq. (3) for $U(\tau )$ and imposing the initial condition $\langle \sigma _{x}(t=0) \rangle =1$, for the detected spin mean value parallel to the *y* axis we obtain

12 $$\begin{aligned} \begin{aligned} \langle \psi | \sigma _{y}(\tau )| \psi \rangle&= |\langle \psi | \lambda _{+} \rangle |^2 \langle \lambda _{+} | \sigma _{y}^{0} | \lambda _{+} \rangle + |\langle \psi | \lambda _{-} \rangle |^2 \langle \lambda _{-} | \sigma _{y}^{0} | \lambda _{-} \rangle \\&\quad + 2 {{\,\mathrm{\displaystyle Re}\,}}\bigg ( {{\,\mathrm{\displaystyle e}\,}}^{i \Omega _{L} \tau } \langle \psi | \lambda _{+} \rangle \langle \lambda _{+} | \sigma _{y}^{0} | \lambda _{-} \rangle \langle \lambda _{-} | \psi \rangle \bigg ) \\&\quad + 2 {{\,\mathrm{\displaystyle Re}\,}}\sum _{s>0} \bigg [ {{\,\mathrm{\displaystyle e}\,}}^{i s \tau }\, |\langle \psi | \lambda _{+} \rangle |^2 \langle \lambda _{+} | \sigma _{y}^{s} | \lambda _{+} \rangle + {{\,\mathrm{\displaystyle e}\,}}^{i s \tau }\, |\langle \psi | \lambda _{-} \rangle |^2 \langle \lambda _{-} | \sigma _{y}^{s}| \lambda _{-} \rangle \\&\quad + {{\,\mathrm{\displaystyle e}\,}}^{i (s+\Omega _L) \tau }\, \langle \psi | \lambda _{+} \rangle \langle \lambda _{+} | \sigma _{y}^{s} | \lambda _{-} \rangle \langle \lambda _{-} | \psi \rangle + {{\,\mathrm{\displaystyle e}\,}}^{i (s-\Omega _L) \tau }\, \langle \psi | \lambda _{-} \rangle \langle \lambda _{-} | \sigma _{y}^{s} | \lambda _{+} \rangle \langle \lambda _{+} | \psi \rangle \bigg ], \end{aligned} \end{aligned}$$

with $|\psi \rangle$ the state of interest. For the 0-th $\Omega _L$ component and for the *s*-th harmonic of the micromotion, the introduced $\sigma _{y}^{s}$ Pauli matrix is defined as

13 $$\begin{aligned} \sigma _{y}^{s}=\sum _m P_{m-s}^\dagger \sigma _y P_m, \end{aligned}$$

with the $P_n$ operator introduced in Eq. (8). The $\langle \sigma _y(\tau ) \rangle$ time dependence includes a constant term (first line), a time periodic evolution at the $\Omega _L$ dressed Larmor frequency (second line) leading to the phase shift periodic dependence of Eq. (11). These components are superimposed on the micromotion evolution at the *s*-th harmonic of the dressing frequency (third line) and, finally, the dressed-frequency micromotion interplay at the sidebands $s\pm \Omega _L$ (fourth line). Note that in the ion-cooling community the above *s*-th harmonic micromoton is denoted as “sideband”.

The Qubit time evolution section of the Supplemental Information^48^ contains a perturbation analysis of the $\langle \sigma _{y}(\tau ) \rangle$ time evolution leading to an alternative derivation of phase shifted sinusoidal evolution of Eq. (11).

### Experimental protocol

#### Set-up

In the experimental setup of Ref.^38^, an $^{85}$Rb atom sample is trapped in a Magneto-Optical Trap (MOT), laser-cooled in the $F_g=3$ hyperfine state to few tens $\mu$K. Atoms are then released and spin-polarized along the *x* axis by circularly-polarized pump laser in presence of an uniform magnetic field, with main component $B_{0z}$ and eventually small $B_{0x},B_{0y}$ components. At the end of the polarization phase two radio-frequency linearly-polarized magnetic fields, in the 20–150 kHz range with amplitudes in the 0–50  $\upmu$T range, are applied along the *x* and *y* directions to the released, polarized atoms. In our data the time scale of the *x* dressing evolution corresponds to the $\Phi _{0x}=0$ choice. Starting from the initial $\langle \sigma _x(t=0) \rangle =1$ magnetization, the $\langle \sigma _y(t)\rangle$ magnetization is probed by a linearly polarized beam, propagating along the *y* direction, by detecting the Faraday rotation. The initial preparation and the time evolution detection are detailed in the Supplemental Information^48^. Also the compensation of spurious static magnetic fields and the calibration of the dressing radiofrequency fields are described there.

#### RPP: resonant pulsed pumping

In this configuration, a $5\,\upmu$s laser pulse, periodic at $f_{RPP}$ frequency, forces the atoms into the $\langle \sigma _x(0) \rangle = 1$ state. The Faraday rotation output signal is detected through a lock-in procedure.

Amplitude and phase of the lock-in signal are recorded as a function of $f_{RPP}$. This configuration, corresponding to a Bell and Bloom magnetometer with synchronous optical pumping, has a high sensitivity. We assume that the pump laser does not disturb in a significant way the dynamics of the qubit, but has only the effect of zeroing the dissipative processes allowing to treat the problem within a Hamiltonian formalism. The RPP resonance linewidth is $\approx 600\,$ Hz HWHM and the central resonance frequency is measured with a $50\,$ Hz precision. This spin probe is analogue to the self-sustaining Larmor precession signal detected in the magnetometer experiments of Refs.^54, 55^.

#### DFE: dressed free evolution

In this configuration a single 200 $\upmu$s long pulse of the pumping laser applied in zero static field condition, aligns the qubit spins along the *x* axis. At the end of the pumping phase, with static and dressing fields switched on, the qubit precession is detected by the Faraday rotation. This configuration, with a lower sensitivity than the RPP, detects very precisely amplitude and phase of the qubit time components. A similar pulsed approach with dressing fields on and a pulsed magnetic fields was applied in Ref.^35^.

#### FFT: FFT spectra

An FFT analysis of the time evolution of the spin magnetization is performed in both RPP and DFE modes. The FFT is digitally computed on a time sequence of $\approx 5\,$ms duration at a 500 kHz sampling rate, corresponding to a FFT frequency span of $250$ kHz with a $200$ Hz frequency step. In the RPP case a component at the pump pumping frequency is always present in the spectrum, and the free evolution spectra are examined only for a resonant driving. The information gathered by the two cases produces identical spectral components. In the DFE case though, the spectral lines are larger due to the damping of the atomic magnetization. In both RPP and DFE methods the high-frequency components have a reduced amplitude due to the limited bandwidth of our detector.

### Supplementary Information

Supplementary Information.

## Data Availability

The datasets used and/or analysed during the current study are available from the corresponding author on reasonable request.
