# Supplementary material for: Floquet space exploration for the dual-dressing of a qubit
Source: Sci Rep. 2023 Sep 18;13:15304. doi: 10.1038/s41598-023-41693-2 (PMC10507086; doi:10.1038/s41598-023-41693-2)
Supplement: Supplementary file 1 — Supplementary Information. [file 41598_2023_41693_MOESM1_ESM.pdf]

Supplementary Information: Floquet space exploration for the dual-dressing of a qubit

Alessandro Fregosi[1,2], Carmela Marinelli[2,1], Carlo Gabbanini[1], Giuseppe Bevilacqua[2], Valerio Biancalana[2], Ennio Arimondo[3,1], and Andrea Fioretti[1,*]

[1]Istituto Nazionale di Ottica, CNR-INO, Via G. Moruzzi 1, 56124 Pisa, Italy
[2]Dip. di Scienze Fisiche, della Terra e dell'Ambiente, Università degli Studi di Siena, Via Roma 56, 53100 Siena, Italy
[3]Dipartimento di Fisica, University of Pisa, Largo Bruno Pontecorvo 3, 56127 Pisa, Italy
[*]corresponding author: andrea.fioretti@ino.cnr.it

Introduction

This complement to the Main Text[1] includes two separate sections, a theoretical and an experimental one. The first one begins with a perturbation treatment of the dressed system response. This approach produces an alternative derivation of the experimentally monitored $\langle\sigma_y(\tau)\rangle$ time dependence, not strictly valid for the strong dressings of the experimental investigation. However this perturbation approach provides a simplified interpretation of the data. The theory section continues with a derivation of the discontinuity for the dynamical phase shift associated with the stroboscopic response of the dual-dressed qubit. The experimental section describes in some detail the setup and the geometry of the magnetic fields.

Theory

Kick operator. The dressing operation modifies mean value and time evolution of the spin components, as for the $\langle\sigma_y(t)\rangle$ expectation value described by Eq. (12) of the Main Text. That equation allows the theoretical reproduction of the measured qubit global time evolution described there. For an alternative analysis of the qubit time evolution, we report here the qubit time dependence derived in a perturbation treatment valid for $\Omega_x \gg |\boldsymbol{\omega}_0|, \Omega_y$[2] based on components of the $\mathscr{K}$ kick operator introduced by Eq. (4) of the Main Text.

The $\mathscr{K}$ kick operator, written as a function of the qubit spin operators, is given by

$$\mathscr{K}(\tau) = (K_x(\tau)\sigma_x + K_y(\tau)\sigma_y + K_z(\tau)\sigma_z)/2 = \boldsymbol{K}(\tau)\cdot\boldsymbol{\sigma}/2 = \kappa(\tau)\widehat{\boldsymbol{K}}(\tau)\cdot\boldsymbol{\sigma}/2. \tag{1}$$

where the K_i functions $(i = x,y,z)$ are periodic, $K_i(\tau) = K_i(\tau+2\pi)$, and satisfy the boundary conditions $K_i(0) = 0$ required to enforce $U(0) = \mathbb{1}$ in Eq. (5) of the Main Text. The introduction of the modulus $\kappa(\tau) = |\boldsymbol{K}(\tau)|$ and of the versor $\widehat{\boldsymbol{K}} = \boldsymbol{K}/\kappa$, allows to write

$$\mathrm{e}^{-i\mathscr{K}} = \cos(\kappa/2)\mathbb{1} - i\sin(\kappa/2)\widehat{\boldsymbol{K}}\cdot\boldsymbol{\sigma}, \tag{2}$$

and to evaluate the P_n operators of Eq. (8) of the Main Text as

$$P_n \equiv \left(\frac{1}{2\pi}\int_0^{2\pi}\mathrm{e}^{-in\tau}\cos(\kappa/2)\,\mathrm{d}\tau\right)\mathbb{1} - i\left(\frac{1}{2\pi}\int_0^{2\pi}\mathrm{e}^{-in\tau}\sin(\kappa/2)\widehat{\boldsymbol{K}}\mathrm{d}\tau\right)\cdot\boldsymbol{\sigma} \qquad n\in\mathbb{Z}. \tag{3}$$

These operators appear in the construction of the micromotion-dressed Pauli matrices of Eq. (13) of the Main Text.

For FFT parameters presented in Fig. 5 of the Main Text, Fig. 1 reports numerical values for the kick operator time dependent components vs the $\tau = (0,2\pi)$ unitary time of the micromotion evolution. The amplitude of the kick components for two FFT spectra of the Main Text[1] is presented in order to evidence their role. In the strong dressing regime of these plots all the spatial components of the operator are different from zero. The largest harmonic components of the operator determines the micromotion frequency terms appearing into the FFT spectra reported in Fig. 5 of the Main Text.

Qubit time evolution Using the above kick operator components, the qubit $\langle\sigma_y(\tau)\rangle$ time dependence derived within the perturbative treatment results

$$\begin{aligned}\langle\sigma_y(\tau)\rangle =& J_0(\Omega_x)\left[\sin(\Omega_\mathrm{L}\tau) + K_z^{(1)}(\tau)\cos(\Omega_\mathrm{L}\tau)\right]\\ &+2\sum_{s=2,even}^{\infty} J_s(\Omega_x)\left[\sin(\Omega_\mathrm{L}\tau) + K_z^{(1)}(\tau)\cos(\Omega_\mathrm{L}\tau)\right]\cos(s\tau)\\ &+2K_y^{(1)}(\tau)\sum_{s=1,odd}^{\infty} J_s(\Omega_x)\cos(\Omega_\mathrm{L}\tau)\sin(s\tau)\,.\end{aligned} \tag{4}$$

The structure of this solution is equivalent to that of Eq. (12) of the Main Text, except for the absence of static component and of the sidebands of the micromotion harmonics. The first line describing the Ω_L precession contains the $\sin(\Omega_L t)$ 0-th order evolution, and also a first-order $K_z^{(1)}$ kick operator contribution, $\pi/2$ phase shifted from the previous one. Their combination produces the sinusoidal Ω_L evolution modified and phase shifted by the micromotion presence as described by Eq. (11) of the Main Text.

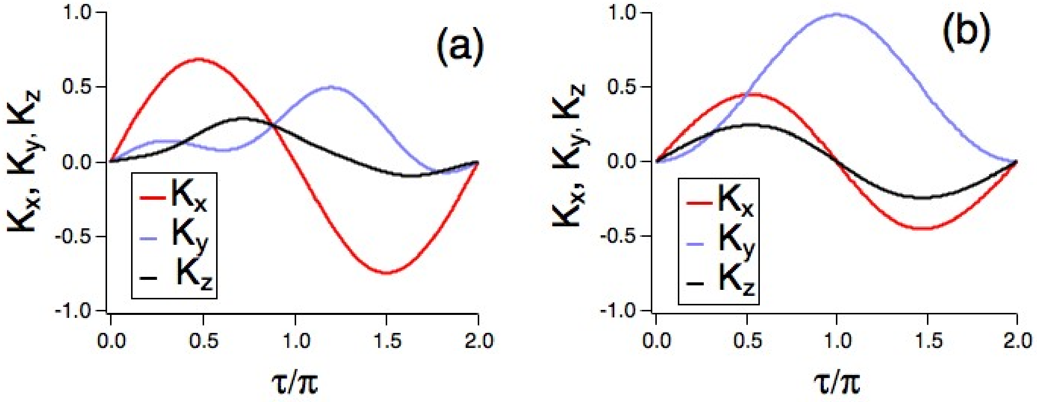

Figure 1. Numerical simulation of the kick operator components in the time interval $(0,2\pi)$. In the strong dressing regime of these plots all three spatial components of the operator are different from zero. Parameters $[p,\Omega_x,\Omega_y,\Delta\Phi_0/\pi,f_L]$ the last one in kHz: in (a) [2,1. 40, 0.55, 0.0, 8.8]; in (b) [1, 1.00, 1.00, 1.5, 8]. In all plots $\omega_{0x}=\omega_{0y}=0$, $\omega_{0z}=0.3375$, and $f=40.00$ kHz. corresponding to those in Figs 5(b) and 5(d), respectively, of the Main Text.

Phase shift discontinuity . The phase shift discontinuities reported in Fig. 6(c) of the Main Text for the $\Delta\Phi_{0,D}$ dressing phase Dirac points are here linked to the discontinuous nature of the Λ qubit projectors. We derive a theoretical expression for those phase shift jumps.

For a dressing phase $\Delta\Phi_0$ close to the Dirac point, the $\boldsymbol{h}$ effective magnetic field introduced in Eq. (9) in[1] crosses the zero value linearly. We introduce a vector $\boldsymbol{\alpha}$ characterizing that crossing as

$$\boldsymbol{h}\approx\boldsymbol{\alpha}(\Delta\Phi_0-\Delta\Phi_{0,D})=\boldsymbol{\alpha}\,\varepsilon \tag{5}$$

where ε denotes the small distance from the Dirac point. This equation evidences the presence of discontinuity in the stroboscopic operators. In fact, using the Λ to $\boldsymbol{h}$ connection of Eq. (9) in[1] the Λ projectors are written as

$$|\lambda_\pm\rangle\langle\lambda_\pm|=\frac{1}{2}\mathbb{1}\pm\frac{1}{2}\frac{\boldsymbol{h}\cdot\boldsymbol{\sigma}}{|\boldsymbol{h}|}=\frac{1}{2}\mathbb{1}\pm\frac{1}{2}\operatorname{sign}(\varepsilon)\frac{\boldsymbol{\alpha}\cdot\boldsymbol{\sigma}}{|\boldsymbol{\alpha}|}. \tag{6}$$

We concentrate our attention on the the first line of Eq. (4), or the equivalent Eq. (12) in[1], describing the complex amplitude of the Ω_L qubit precession in the $\langle\sigma_y(\tau)\rangle$ signal. That amplitude is denoted as $\mathscr{A}$ in the following. We verified numerically that the operator σ_y^0 appearing in that equation is continuous through the Dirac point. Therefore it can be written as $\sigma_y^0=\boldsymbol{\beta}\cdot\boldsymbol{\sigma}/2$ for some β_i with $(i=x,y,z)$. After some algebra one finds for the $\mathscr{A}$ amplitude the following expression:

$$\mathscr{A}\equiv\langle\psi|\lambda_+\rangle\langle\lambda_+|\sigma_y^0|\lambda_-\rangle\langle\lambda_-|\psi\rangle=\left(\frac{3}{8}\frac{(\boldsymbol{\beta}\times\boldsymbol{\alpha})\times\boldsymbol{\alpha}}{\alpha^2}-i\operatorname{sign}(\varepsilon)\frac{\boldsymbol{\beta}\times\boldsymbol{\alpha}}{2\alpha}\right)\cdot\langle\psi|\boldsymbol{\sigma}|\psi\rangle. \tag{7}$$

It follows

$$\mathscr{A}(\varepsilon=0^+)-\mathscr{A}(\varepsilon=0^-)=\mathscr{A}_{0^+}-\mathscr{A}_{0^-}=-i\frac{\boldsymbol{\beta}\times\boldsymbol{\alpha}}{\alpha}\cdot\langle\psi|\boldsymbol{\sigma}|\psi\rangle, \tag{8}$$

where the first equality represents a compact definition. Therefore

$$\mathscr{A}_{0^+}=\mathscr{A}^*_{0^-}\qquad\text{and}\qquad\mathscr{A}_{0^\pm}=|\mathscr{A}|\,\mathrm{e}^{\pm i\theta_0}\,. \tag{9}$$

The $2\theta_0$ phase difference corresponds to the phase jump visible in the theoretical and experimental results of Fig. 6(c) of the Main Text.

Experimental protocol

Set-up. In the experimental setup, described in more detail in ref.[3], a sample of ^{85}Rb atoms is trapped in a Magneto-Optical Trap (MOT), laser-cooled in the $F_g = 3$ hyperfine state to a final temperature of several tens microkelvin. Atoms are then released and spin-polarized along the x axis in a uniform magnetic field, with main component B_{0z} and eventually an additional, small B_{0x}, B_{0y} components. The atomic spin polarization is produced by a circularly-polarized pump laser pulse, $10-100\mu$s long, propagating along the x direction. The pumping laser is tuned on the $F_g = 3 \rightarrow F_e = 3$ ^{85}Rb D_2 transition. At the low magnetic fields of the experiment, around $3\,\mu$T, the atomic multilevel system is equivalent to a two-level system. At the end of the polarization phase two radio-frequency linearly-polarized magnetic fields, in the 20-150 kHz range with amplitudes in the $50\,\mu$T range, are applied along the x and y directions to the released, polarized atoms.

Starting from the initial $\langle\sigma_x(t=0)\rangle = 1$ atomic magnetization, the qubit performs a precession at the f_L Larmor frequency

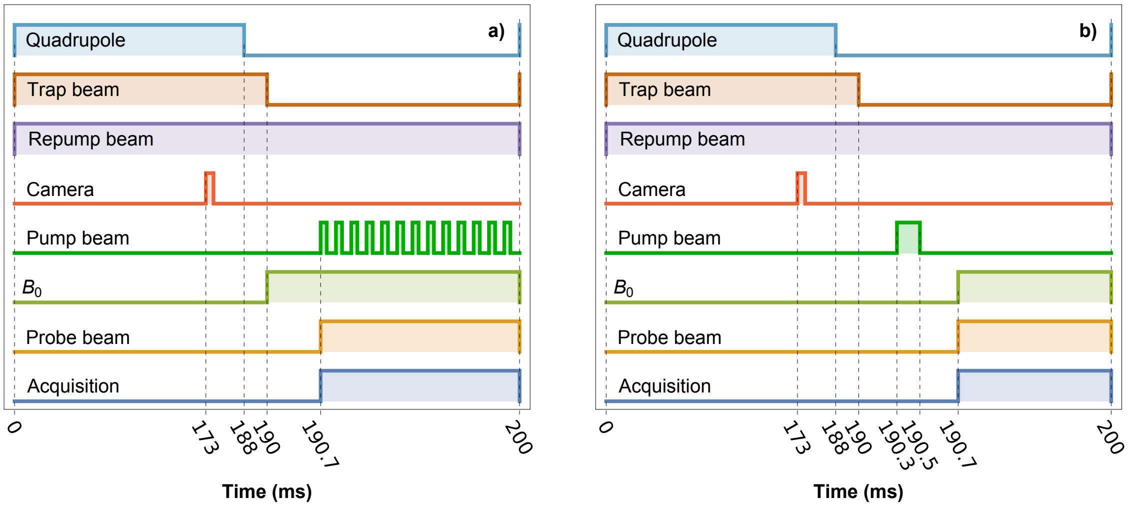

Figure 2. Experimental time sequence for (a) the Resonant Pulsed Pumping (RPP) mode and (b) the Dressed Free Evolution (DFE) mode of the qubit spin, respectively. The first three lines (Quadrupole field of the MOT, trap laser, repump laser and camera) concern the cold sample preparation in the MOT and the measure of the number of atoms, while the other lines the dressed spin evolution and the signal acquisition.

around the effective magnetic field produced by the combined action of static and radio-frequency magnetic fields. A linearly polarized laser, blue-detuned by 320 MHz with respect to $F_g = 3 \rightarrow F_e = 4$ transition and propagating along the y direction probes the $\langle\sigma_y(t)\rangle$ magnetization on the basis of the detection of the Faraday rotation. The polarization of the transmitted probe laser is analyzed by a balanced polarimeter, with the difference output signal proportional to the polarization rotation angle of the transmitted probe beam. The polarimeter signals are digitized by a multifunction I/O device (National Instruments, USB-6346, 500 kS/s). The whole experimental sequence is PC controlled through the LabView software. In all acquisitions the time scale of the x dressing evolution corresponds to the $\Phi_{0x} = 0$ choice.

Because the MOT resonant cw lasers and magnetic field gradient are not compatible with the dressed-atom operation, the experiment operates in cycles with a typical 5 Hz repetition rate, 190 ms MOT loading and 10 ms spin evolution. The two experimental sequences, resonant pulsed pumping (RPP) and dressed free evolution (DFE), applied to probe the qubit are depicted in Fig. 2(a) and (b) respectively. A CCD camera, triggered at the end of the MOT loading period, monitored the number of trapped atoms through the atomic fluorescence signal. The magnetization evolution is monitored by the Faraday rotation during the 10 ms free fall time of the cold atomic sample. The atomic spin precession experienced a damped evolution with damping time $\tau_{damp} \approx 1.6$ ms produced by the inhomogeneity of the radiofrequency fields, as described in ref.[4].

Spatial orientation of static and radiofrequency fields. The dual dressing Hamiltonian of[1] accounts for two dressing fields and the static $\boldsymbol{B}_0$ magnetic field, oriented along the (x, y, z) axes, respectively. The presence of weak components of the dressing field along the z axis does not modify the qubit evolution as pointed out in the Supplemental Material of [5]. Note that the above single dressing calibration of the dressing fields determines only their components orthogonal to the z axis. The precise orthogonality of the two dressing fields in the (x, y) plane is not required. As shown in ref.[5] in the perturbation regime a component of the second dressing field parallel to the first one does not modify the dual-dressing response. This behaviour is confirmed by the comparison of the $p = 1$ Ω_L data in Fig. 2(b) (of the Main Text) for the $\Delta\Phi_0 = 0$ and $\Delta\Phi_0/\pi = 1$. For those values the dressing field is linearly polarized, and the qubit response is described by the J_0 single dressing response. For perfectly orthogonal dressing fields, the Ω_L values at $\Delta\Phi_0/\pi = 0$ and $\Delta\Phi_0/\pi = 1$ should be identical. For the Fig. 2(b) data

their difference is within the 0.1 percent error bar. This result with the addition of the the precision in the $\Delta\Phi_0$ determination confirms the negligible role of any not-orthogonal component of dressing field.

References

1. Fregosi, A. et al. Floquet space exploration for the dual-dressing of a qubit. Sci. Reports **??**, ?? (2023).

2. Bevilacqua, G., Biancalana, V., Zanon-Willette, T. & Arimondo, E. Harmonic dual dressing of spin-1/2 systems. Phys. Rev. A **105**, 022619, DOI: 10.1103/PhysRevA.105.022619 (2022).

3. Fregosi, A. et al. Magnetic induction imaging with a cold-atom radio frequency magnetometer. Appl. Phys. Lett. **117**, 144102, DOI: 10.1063/5.0020415 (2020). https://doi.org/10.1063/5.0020415.

4. Augustine, M. & Hahn, E. Radiation damping with inhomogeneous broadening: Limitations of the single Bloch vector model. Concepts Magn. Reson. **100**, 1–7 (2001).

5. Bevilacqua, G., Biancalana, V., Vigilante, A., Zanon-Willette, T. & Arimondo, E. Harmonic fine tuning and triaxial spatial anisotropy of dressed atomic spins. Phys. Rev. Lett. **125**, 093203, DOI: 10.1103/PhysRevLett.125.093203 (2020).
